# Doxorubicin induced immune abnormalities and inflammatory responses via HMGB1, HIF1-α and VEGF pathway in progressive of cardiovascular damage

**DOI:** 10.1016/j.amsu.2022.103501

**Published:** 2022-03-21

**Authors:** Ahmad Syukri, Budu, Mochammad Hatta, Muzakkir Amir, Mohammad Saifur Rohman, Idar Mappangara, Cahyono Kaelan, Siti Wahyuni, Agussalim Bukhari, Ade Rifka Junita, Muhammad Reza Primaguna, Ressy Dwiyanti, Andini Febrianti

**Affiliations:** a Post Graduate School. Faculty of Medicine, Hasanuddin University, Makassar, Indonesia; b Department of Ophthalmology, Faculty of Medicine Hasanuddin University, Makassar, Indonesia; c Molecular Biology and Immunology Laboratory, Faculty of Medicine, Hasanuddin University, Makassar, Indonesia; d Department Cardiology and Vascular Medicine, Faculty of Medicine Hasanuddin University, Makassar, Indonesia; e Department of Cardiology and Vascular Medicine, Faculty of Medicine, Saiful Anwar General Hospital, Brawijaya University, Malang, East Java, Indonesia; f Department of Pathology Anatomy, Faculty of Medicine, Hasanuddin University, Makassar, Indonesia; g Department of Parasitology, Faculty of Medicine, Hasanuddin University, Makassar, Indonesia; h Department of Nutrition, Faculty of Medicine, Hasanuddin University, Makassar, Indonesia; i Department of Internal Medicine, Faculty of Medicine, Hasanuddin University, Makassar, Indonesia; j Department of Medical Microbiology, Faculty of Medicine, Tadulako University, Palu, Indonesia; k Department of Forensic and Medicolegal, Faculty of Medicine, Hasanuddin University, Makassar, Indonesia

**Keywords:** DOX, HMGB1, HIF-1α, VEGF, Cardiotoxicity, Cardiovascular disease

## Abstract

**Background:**

Doxorubicin (DOX) is a commonly used treatment for cancer and the mechanism of DOX-induced cardiomyocyte damage in cardiovascular disease is not fully understood. High-mobility group box 1 (HMGB1), strong induce proinflammatory cytokines via damage associated molecular pattern (DAMP) which its interaction with the receptor of advanced glycation end products (RAGE), that affect cytokine release, and angiogenesis via the role of HMBG1, HIF-1α and VEGF as an important regulator in these cardiac failure processes. Hypoxia-inducible factor-1α (HIF-1α) is plays an important role in the cellular response to systemic oxygen levels of cells and VEGF is an angiogenic factor and can stimulate cellular responses on the surface of endothelial cells will be described

**Objective:**

The aim of this article is to comprehensively review the role of HMGB1, HIF-1α, and VEGF in DOX-induced Cardiovascular Disease and its molecular mechanisms.

**Methods:**

The data in this study were collect by search the keyword combinations of medical subject headings (MeSH) of “HMGB1”, “HIF-1 α”, “VEGF”, “DOX” and “Cardiovascular disease” and relevant reference lists were manually searched in PubMed, EMBASE and Scopus database. All relevant articles in data base above were included and narratively discussed in this review article.

**Results:**

Several articles were revealed that molecular mechanisms of the DOX in cardiomyocyte damage and related to HMGB1, HIF-1α and VEGF and may potential treatment and prevention to cardiovascular disease in DOX intervention.

**Conclusion:**

HMGB1, HIF-1α and VEGF has a pivotal regulator in DOX-induce cardiomyocyte damage and predominantly acts through different pathways. The role of HMGB1 in DOX-induced myocardial damage suggests that HMGB1 is a mediator of DOX-induced damage. In addition, DOX can inhibit HIF-1α activity where DOX can decrease HIF-1α expression and HIF-1α is also responsible for upregulation of several angiogenic factors, including VEGF. VEGF plays an important role in angiogenesis and anti-angiogenesis both in vitro and in vivo and reduces the side effects of DOX markedly. In addition, the administration of anti-angiogenesis will show an inhibitory effect on angiogenesis mediated by the VEGF signaling pathway and triggered by DOX in cells.

## Highlights

• The effect of Doxorubicin (DOX) induced cardiovascular damage via several pathways.
• Cardiovascular damage can involve HMGB1, HIF-1α, and VEGF.
• HMGB1, HIF-1α, and VEGF as a pivotal regulator in DOX-induce cardiomyocyte damage.
• HMGB1, HIF-1α, and VEGF in cardiovascular diseases will be predominantly acting through different pathways.

## 1 Introduction

Doxorubicin (DOX) is known to cause cardiotoxicity through several common mechanisms, such as oxidative stress, DNA and mitochondrial damage, and iron accumulation, as well as through novel pathways such as induction of autophagy and CYP1. However, each pathway and how these pathways interact with each other is not well understood, so new approaches to cardiac protection are needed. The pathomechanism of cardiotoxicity in DOX administration is an opportunity in the prevention and treatment of heart failure [1].

High mobility group box 1 (HMGB1) is a protein that in humans is encoded by the HMGB1 gene located on chromosome 13 [[2], [3], [4]]. High mobility group box 1 protein is secreted by immune cells such as macrophages, monocytes, and dendritic cells via a secretory pathway. In the inflammatory process, activated macrophages and monocytes will release HMGB1 as a cytokine mediator [5,6]. HMGB1 binds to nuclear DNA, which is then actively released in the presence of cytokine stimulation and passively during cell injury and death. It was reported that HMGB1 is one of the sensors of autophagy with oxidative stress. Inhibition of HMGB1 release reduces the number of autolysosomes and “autophagic flux” in human and mouse cell lines under oxidative stress such as infection condition, and traumatic or injury [[7], [8], [9]]. This situation provides an overview of the role of HMGB1 and Damage Associated Molecular Pattern (DAMP) in triggering autophagy as a cell defense mechanism under stress conditions [[10], [11], [12]]. HMGB1 is involved in the regulation of transcription, DNA replication and repair, as well as nucleosome formation. HMGB1 is passively released by necrotic tissue or actively secreted by cells under induced stress. Extracellular HMGB1 acts as a DAMP and gives rise to several redox forms that by binding to various receptors and interactors promote various cellular responses, including inflammation or tissue regeneration [13,14]. Inhibition of extracellular HMGB1 in experimental models of myocardial ischemia/reperfusion injury, myocarditis, cardiomyopathy induced by mechanical stress, diabetes, bacterial infection or chemotherapy drugs reduces inflammation and is protective. In contrast, administration of HMGB1 after myocardial infarction induced by permanent coronary artery ligation improves cardiac performance by promoting tissue regeneration. HMGB1 decreases contractility and induces hypertrophy and apoptosis in cardiomyocytes, stimulates cardiac fibroblast activity, and promotes the proliferation and differentiation of cardiac stem cells. High levels of HMGB1 will protect cardiomyocytes from apoptosis by preventing DNA oxidative stress, and in mice with overexpression of HMGB1 will protect cardiomyocytes from damage to the heart. Higher circulating HMGB1 is associated with human heart disease. In cardiac injury, HMGB1 elicits a beneficial response that may depend on the formation and stability of various redox forms, the specific function of which in this relationship remains unclear. However, HMGB1 on cardiac dysfunction has potential as therapeutic in modulating oxidative expression and activity [15,16].

HMGB1 can interact with Toll-like receptor (TLR) ligands and cytokines, and activate cells via several surface receptors including TLR2, TLR4, and RAGE. Molecular mechanisms reveal that HMGB1 binding and TLR4 signaling mediate cytokine release and tissue damage including caused infectious diseases [17,18]. Previous studies has been showed that the role of HMGB1 in DOX-induced myocardial damage and showed that HMGB1 is a mediator of DOX-induced damage [19]. Hypoxia inducible factor 1α (HIF-1α) is a subunit of the hypoxia-induced heterodimeric transcription factor 1 (HIF-1α) encoded by the HIF-1α gene which in humans is located on chromosome 14q [20,21].

Hypoxia-induced HIF-1α gene, a transcription factor important for cardiovascular development and systemic O2 homeostasis [22,23], whereby dysregulation and overexpression of HIF-1α in hypoxic states or genetic alterations has been heavily implicated in cancer biology, as well as a number of other pathophysiologies particularly in the areas of vascularization and angiogenesis, energy metabolism, cell survival, tumor invasion, injury and other diseases via caspase 3, nitrate oxidase, Peroxisome Proliferator-Activated Receptor Gamma Coactivator 1- alpha (PGC-1α) or oxidative stress [[24], [25], [26], [27], [28], [29]]. The accumulation of HIF-1α due to DOX induction in cells is caused by increased expression and activation of Signal transducer and activator of transcription 1 (STAT1) where this activation will stimulate the expression of iNOS and its NO synthesis in tumor cells. In this study, suppression of NO synthesis or STAT1 activation will reduce the accumulation of HIF-1α induced by doxorubicin (DOX) in cancer cells, where DOX chemotherapy can induce HIF-1α accumulation in cells and is a condition that will limit the efficacy of DOX therapy [30]. Another study, which was related to the incidence of hypoxia, showed that administration of nanoparticle polymers containing DOX could inhibit HIF-1α activity in patients with Age-related macular degeneration (AMD). Retinal pigment epithelia (ARPE-19) cell line. However, besides that HIF-1α is also responsible for the upregulation of several angiogenic factors, including VEGF [31]. Several studies on the mechanism and relationship of DOX with HIF-1α and VEGF expression are still unclear, which in this study showed that doxorubicin can suppress the induction of hypoxia due to VEGF expression by inhibiting HIF-1α through different mechanisms [32].

Vascular endothelial growth factor (VEGF) is a protein found in humans that is encoded by a gene located on chromosome 6p. VEGF functions to stimulate the formation of new blood vessels and increase blood capillary flow. VEGF is a polypeptide, where VEGF deficiency is quite common and is a serious problem in patients with biotoxin disease that must be corrected. VEGF is a substance made by cells that stimulates the formation of new blood vessels and increases blood capillary flow in several diseases [[33], [34], [35], [36], [37], [38]]. VEGF is a polypeptide and impaired VEGF function is a serious problem in patients in a toxic state [39]. VEGF plays an important role in angiogenesis and is highly visible in carcinomas, where it is currently an important target for cancer therapy. Previous studies have shown that administration of anti-neuroblastoma and anti-angiogenesis both in vitro and in vivo, and significantly reduced the side effects of DOX, especially liver damage. In addition, administration of anti-angiogenesis will show an inhibitory effect of angiogenesis mediated by the VEGF signaling pathway and triggered by DOX in cells [40]. Cardiovascular toxicity associated with anti-VEGF agents has become a major risk in cancer treatment, where suppression of VEGF expression, through alterations in Nitric Oxyde (NO) suppression and endotheline-1 (ET-1) stimulation, determines endothelial dysfunction and vasoconstriction, leading to is the main reason for hypertension. Research underlying cardiovascular toxicity can help determine the overlapping mechanisms between cardiovascular disease and existing or future drug toxicity [41].

Based on the background and previous findings, a study to more completely will be urgent to understand in the effect of induction of Doxorubicin (DOX) on changes in HMBG1, HIF-1α and VEGF which may be a mechanism for cardiovascular disorders.

## 2 Method

A comprehensive search of literature was conducted in PubMed (NIH), Scopus, EMBASE, and Google Scholar database using keyword combinations of the medical subject headings (MeSH) of “HMGB1”, “HIF-1α”, “VEGF”, “DOX” and “Cardiovascular disease” and relevant reference lists were also manually searched. The review was preferred Reporting Items for Systematic Reviews and Meta-Analyses Protocols (PRISMA) criteria also has been followed [42] and AMSAR2 [43]. The review was registered in www.researchregistry.com with unique identifying number: reviewregistry966 [44]. All relevant articles, with English language, of any study design published in data base above were included and narratively discussed.

## 3 Results

### 3.1 High mobility group box protein I (HMGB1)

Angiogenesis is an important initial step in the growth and proliferation of cells with adequate amounts of oxygen. The rapid cell growth is accompanied by a reduced density of micro-vessels, resulting in chronic hypoxia that often leads to necrotic areas of the cells. These hypoxic and necrotic areas show increased expression of angiogenetic growth factors, for example, vascular endothelial growth factor (VEGF) and can also attract macrophages, which secrete a number of potent angiogenetic cytokines and growth factors. A number of molecules that can act as mediators of angiogenesis are High mobility group box I (HMGB1). Recent studies have shown that HMGB1, known as an architectural chromatin-binding protein, can be released extracellularly by passive diffusion from activated necrotic cells and macrophages [45].

To examine the angiogenetic effect of HMGB1 on endothelial cells, an in vitro spheroid model was used. The results showed that exogenous administration of HMGB1 induces endothelial cell migration and proliferation in vitro and proved that endothelial cells can be induced by HMGB1 in vitro [[46], [47], [48], [49]]. Previous studies have shown that HMGB1 from macrophage cells is expressed in vitro 8 h after stimulation with endotoxin, TNF, or IL1 and an increase in serum HMBG1 levels occurs 8–32 h after endotoxin exposure [[50], [51], [52]]. Recombinant HMGB1 was shown to increase DOX resistance and this was associated with evidence of an autophagy process. Potential substances secreted by breast cancer associated fibroblasts (BCFs) will induce intracellular HMGB1 expression in breast cancer cells. After exposure to doxorubicin (DOX), there is an increase in extracellular HMGB1 and this HMGB1 will function in a paracrine manner to induce chemoresistance of cancer cells that survive nearby. In addition, recombinant HMGB1 was shown to increase DOX resistance and this was associated with evidence of autophagy [50]. HMGB1 is a chromatin-associated core protein as well as an extracellular molecule of damage associated molecular patterns (DAMP) which can be found in a number of hypoxic states, including the effects of toxic agents, tumors, traumatic injury [[53], [54], [55]]. Several chemotherapeutic agents used in cancer treatment including DOX can induce the release of HMGB1 into the tumor environment after cell death and suggest that neutrophil cells and macrophages will be activated by cytokines as part of the innate immune response against cancer cells and will actively excrete HMGB1. Stromal fibroblast cells in breast cancer may also play a similar role in chemoresistance through upregulation of HMGB1 in cancer cells during chemotherapy-mediated cell death [56].

Due to damage, stress and inflammatory mediators that can be caused by toxic substances to cells, HMGB1 will be expressed both intracellularly and extracellularly with end results involved some cytokines in infection condition [[57], [58], [59], [60], [61], [62], [63]], metabolic disorder [64], allergy [65,66] and traumatic injury [[67], [68], [69], [70]]. Expression of HMGB1 will cause an inflammatory process and affect the occurrence of ROS which will increase autophagy [56]. With the combination of the above processes, there is the release of extracellular vesicular (EV) calcifin and will unite with the extracellular matrix, which will eventually form calcium deposits in the cardiovascular system, including atherosclerotic calcification and calcification of aortic valve disease, ischemic heart. Although cardiovascular calcification has long been considered a passive degenerative event, it is now recognized as an active and highly regulated process involving osteochondrogenic differentiation, apoptosis, and release of extracellular vesicles. Although there have been many studies on the pathogenesis of cardiovascular calcifications, the underlying mechanism remains unclear [71]. HMGB1 is a protein found in the cell nucleus and bound to chromatin in almost all eukaryotic cells, where it functions as a damage associated molecular pattern (DAMP) when released into the extracellular space following activation, induction, injury, and cell death. In addition, HMGB1 also functions as an active cytokine on bone cells that participates in bone remodeling and the pathogenesis of ectopic calcifications [72]. However, research on the role of HMGB1 in promoting cardiovascular calcification and its mechanism remains unclear [71].

In ischemic states, the effect of cardiotoxic drugs, hyperglycemia, infection, autoimmune response or mechanical stress causes oxidative stress and nitrosative stress which then causes tissue necrosis with the resultant passive release of HMGB1 or increased secretion of HMGB1 acetate from the heart actively and stimulation of inflammatory cells [73]. Extracellular Fr-HMGB1 undergoes progressive oxidation but an undetermined redox form can exacerbate inflammation and cause cell apoptosis, cardiomyocyte hypertrophy (CM) and activation of cardiac fibroblasts (CF) to produce Collagen. These cell responses determine the development of cardiac hypertrophy and/or fibrosis and ultimately heart failure. Modulation of the oxidative state of HMGB1 could be a strategy to limit inflammation and damage, and support tissue repair. In addition, cardiac injury causes elevated blood levels of HMGB1 and various forms of the modified protein can be associated with different disease stages, and may represent a selective prognostic marker of the degree of cardiac damage and aid in risk stratification [74].

HMGB1 is a chromatin protein in the cell nucleus where when oxidative stress and nitrosative stress occur, HMGB1 is released in heart cells. To date, the role of the redox form of HMGB1 in the context of cardiac injury remains largely unexplored, opening up promising new investigative avenues in this area [75]. Obviously, oxidative and nitrosative stress after insult affects the expression, secretion and release of HMGB1 from cardiac cells. It is likely that differences in the levels of reactive oxygen species (ROS) production during cardiac damage regulate the interconversion kinetics of fr-HMGB1 to ds-HMGB1 and ox-HMGB1, which may partially explain the differences observed after administration of HMGB1 inhibitors in various experimental models of cardiac injury [15]. Fr-HMGB1 and 3S exert opposite effects on cardiac infarction. In an experimental model of myocardial infarction induced by permanent coronary ligation, injection of fr-HMGB1 reduces infarct area and improves cardiac function because it is able to promote angiogenesis and differentiation of resident cardiac stem cells (CPCs) into cardiomyocytes. ROS release after infarction can progressively oxidize fr-HMGB1 to ds-HMGB1 and then to ox-HMGB1, which is important for the regenerative effect of HMGB1. In contrast, injection of a non-oxidable 3S mutant reduces angiogenesis and causes an increase in infarct area and collagen deposition, leading to worsening of cardiac dysfunction [15,76]. HMGB1 will be released passively by necrotic cells, and actively by macrophages/monocytes in response to exogenous and endogenous inflammatory stimuli by PAMPs or DAMP. After binding to the receptor for advanced glycation end products (RAGE) or toll like receptor 4 (TLR4), HMGB1 activates vascular endothelial cells and macrophages/monocytes and T regulation (Treg) to express proinflammatory cytokines, chemokines, and adhesion molecules such as some infectious diseases [[77], [78], [79]] and pregnancy [80] and preeclampsia state [81].

In the inflammatory state of cardiovascular disease, HMGB1 as a proinflammatory cytokine derived from injured endothelium and activated macrophages/monocytes may play a role in atherosclerosis and other cardiovascular disorders [82]. Administration of DOX increases HMGB1 expression and induces TNF- secretion via TLR4, whereas HMGB1 released from necrotic cancer cells treated with necrosis inducers increases regrowth and metastasis of residual cancer cells through RAGE activation [83,84].

### 3.2 Hypoxia inducible Factor-1α (HIF-1α)

Hypoxia inducible factor-1α (HIF-1α) is a transcriptional master regulator expressed in genes that regulate oxygen homeostasis in mammals, including genes involved in erythropoiesis, iron metabolism, angiogenesis, blood flow control, glucose absorption and glycolysis, pH regulation, and cell cycle control. The HIF-1α subunit, regulates the expression of more genes than erythropoietin, and its mechanism depends on cell oxygenation where the basic molecular principle is oxygen sensing [85] Hypoxic states are associated with a variety of physiological and pathophysiological processes, including cell development, wound healing, inflammation, vascular disease, toxins and cancer [24,30,49]. The process of molecular oxygenation of cells through electron acceptors as electron transport chains for the maintenance of oxygen delivery in cells and this is an important process in the bioenergetic homeostasis of cells. Cells will adapt effectively in conditions of disruption of the hemostatic process to hypoxic stress at the molecular level through a transcription factor called HIF-1α [86]. Under normal conditions where there is sufficient oxygen, good hydroxylase occurs and the expression and activation of HIF-1α transcription as oxygen sensing will decrease. Thus, the transcription of hypoxia-responsive HIF-1α is dependent on hydroxylase. Besides that, hydroxylase plays a role in the regulation of Nuclear Factor-κβ (NF-κβ) which regulates the process of the immune system and inflammation and this will be useful in the application of therapy in disorders related to inflammation [87].

The HIF-1α gene consists of 15 exons where transcription of the HIF-1α gene begins in the 15-nt region and in the 0.7 kb region of the 5′ sequence as a promoter that functions in the structure, function and regulation of the HIF-1α gene [88]. Dysregulation and overexpression of HIF-1α in hypoxic conditions of cells have a close relationship with cell pathophysiology, especially vascularization and angiogenesis, cell metabolism and energy, cell survival and death [21,89]. In normoxia, oxygen sensors such as Prolyl Hydroxylase Domain isoforms (PHD1, PHD2, PHD3) and Factor inhibiting HIF-1α (FIH) can hydroxylate HIF-1α on proline 402 and 564 and on asparagine 803. Hydroxylation of prolyl causes poly -ubiquitination and degradation of HIF-1, while hydroxylation of asparaginyl blocks the recruitment of co-activator p300 and CBP-binding protein [CREB (cAMP-response-element-binding protein)] which will inhibit the transactivation function of HIF-1α [87]. Meanwhile, when in a hypoxic state, hydroxylase is inhibited and causes HIF-1α to escape from hydroxylation due to degradation and inactivation of PDH and FIH. This causes HIF-1α to move into the nucleus, and activates HIF-1α gene expression.

In hypoxic conditions, the transcript response is not only influenced by HIF-1α but also has a relationship with the hydroxylase process as an oxygen sensor involving PDH and FIH. Hypoxia is also associated with NF- activity which regulates the main transcriptional processes of gene expression in innate immune and inflammatory cells, both in vitro and in vivo [86]. Previous studies have shown that HIF-1α is associated with upregulation of angiogenic factors, including cardiotoxic VEGF and DOX that can increase the expression of HIF-1α and VEGF. However, by making capsules containing polylactic coglycolic acid (PLGA) and chitosan (NanoDOX) it can suppress the expression of HIF-1α and VEGF in patients with Age-related Macular Degeneration (AMD) [31]. HIF-1α is a major transcription factor that controls cellular homeostasis, which is useful under normal conditions as an oxygenation sensor, but in hypoxic conditions HIF-1α will activate cells associated with oxygenation. This is a major risk factor for the process of angiogenesis, therapeutic resistance and poor prognosis in patients. HIF-1α activity is usually suppressed under normoxic conditions due to oxygen-dependent degradation of HIF-1. Under normoxia, HIF-1α is upregulated in tumor cells in response to DOX as cancer therapy. DOX can also increase VEGF expression by normoxic tumor cells and stimulate tumor angiogenesis. DOX-induced accumulation of HIF-1α in normoxia cells is due to increased expression and activation of Signal Transducer and Activator of Transcription (STAT1), activation that stimulates expression of inducible nitric oxide synthase (iNOS) and synthesis of Nitrite oxide (NO) in cells [90,91].

Previous study showed that inhibiting NO synthesis or STAT1 activation would suppress HIF-1α expression in DOX-induced cells. By knowing the effect of DOX on changes in HIF-1α expression, it can be used as an alternative combination of DOX treatment and can prevent possible cardiotoxic effects in the form of heart failure [30,92]. Other studies have shown that DOX can inhibit the transcriptional activity induced by HIF-1α by blocking its binding to the DNA of experimental animal (rabbit) cells. DOX can also suppress neovascularization (NV) in the choroid and retinal, but also impair retinal function as demonstrated by electroretinograms (ERGs). By using DOX conjugated with polyethylene glycol and poly(sebacic acid) it reduces HIF-1α expression, suppresses choroidal and retinal NV, and does not cause retinal toxicity. The present study concluded that DOX conjugation may be applicable to ocular NV with less toxic effects [93].

### 3.3 Vascular endothelial growth factor (VEGF)

Vascular Endothelial Growth Factor (VEGF) is modulated by Reactive Oxygen Species (ROS) and oxidative stress by stimulating VEGF production in several cell types, including endothelial cells, smooth muscle cells and macrophages. ROS enhance angiogenesis by increasing HIF-1α, as well as VEGF-2 receptor (VEGFR2) expression and activity. The relationship between ROS and VEGF becomes more complex when VEGF promotes cell migration and proliferation by increasing intracellular ROS levels. Moreover, oxidative stress also induces angiogenesis in a VEGF-independent manner by phospholipid oxidation, generating metabolites that act either as ligands or by inducing post-translational modifications e.g. carboxyalkylpyrrole (CAP) proteins in the process of angiogenesis [94,95]. In addition, through the independent VEGF pathway, the process of angiogenesis will involve Toll like receptor (TLR) in infectious diseases [96] and activate NFκB such as in bone demineralization [97]. Previous studies have shown that survivin as an inhibitor of apoptosis family of proteins (IAP) can control cell division, apoptosis, metastasis and excessive angiogenesis in disorders caused by cell hypoxia. The main sources of oxidative stress can come from mitochondria and NADPH oxidase which will produce ROS and trigger angiogenesis through the HIF-1α and VEGF pathways. Also, the angiogenesis pathway can also occur through independent VEGF, namely TLR and NFκB [98].

Proline hydroxylase (PHD) as the main oxygen sensor that mediates the cellular response to hypoxia, where the mitochondria will respond to low oxygen pressure, produces ROS, which then activates intracellular pathways to control the expression of several genes that regulate cell survival. The sensor process for hypoxia in mitochondria will involve ROS to increase HIF-1α expression. In addition, mitochondrial ROS are required for HIF-1 DNA binding and induction of expression of Erythropoietin (EPO), VEGF, as well as HIF-1α-mediated glycolytic enzymes. Thus, mitochondrial ROS play an important role in the stabilization and expression of HIF-1α and VEGF genes under hypoxic conditions [99,100]. Inflammation caused by DOX will increase the expression of NFkB, IL-1β, IL-6, IL-8, TNF- and monocyte chemoattractant protein 1 (MCP-1). In neuroblastoma cells, p53 is required for DOX to activate NFκB. In lung carcinoma Lewis will upregulate IL-1β and IL-6 through the p38/mitogen activated protein kinase (MAPK) pathway. DOX can induce oxorubicin to induce the acquisition of epithelial-mesemchymal transition (eMT) in breast cancer cells [100]. Both VEGF and HIF-1α play an important role in the process of angiogenesis, where the DNA destroying drugs cisplatin and doxorubicin and the microtubule inhibitors docetaxel and paclitaxel can affect VEGF expression and HIF-1α activity in human cells. Previous studies have shown that DOX can suppress the induction of hypoxia, VEGF expression by inhibiting HIF-1α through different mechanisms and these findings may be useful in studying the molecular pathogenesis of hypoxia on DOX administration [32,101].

DOX can cause cardiotoxicity, cardiomyopathy, and congestive heart failure through inflammatory mechanisms, eg by increased prostaglandin E2 and IL-1β. Similarly, several previous studies showed an increase in IL-8, NFκB, TNF-, monocyte chemotactic protein-1 (MCP-1) in DOX-induced rats. Doxorubicin-mediated activation of NFκB and inflammatory cytokines has been demonstrated by the effect of DOX on adipose tissue, thereby increasing serum total cholesterol, triglyceride, and low-density lipoprotein levels. This could be explained by the downregulation of peroxisome proliferator activated receptor gamma induced by DOX in adipose tissue. This is due to a decrease in circulating free fatty acid clearance, an increase in macrophage cells, and activation of NFkB and inflammatory cytokines [98]. Serum VEGF levels are seen to be markedly elevated in metabolic diseases such as POEMS syndrome (polyneuropathy (P), organomegaly (O), endocrinopathy (E), M-protein (M), and skin changes (S) [36]. In neuroblastomas with high VEGF expression and in this study it was shown that SiO2@LDH-Bev can enhance VEGF targeting ability, anti-neuroblastoma and anti-angiogenesis efficiency of DOX. At the same time, SiO2@LDH-Bev-DOX can eliminate cardiac toxicity and liver damage caused by DOX. Thus, the combined administration of Bevacizumab (Bev) and DOX will be safer and more efficient in Neuroblastoma and it is still necessary to study the mechanism of nanoDOX carrier function in cancer therapy both in vitro and in vivo [40].

Cardiovascular toxicity due to DOX administration, which has become a risk in cancer treatment, can be attributed to anti-VEGF agents where suppression of VEGF expression, through changes in NO inhibition and endothelin stimulation (ET1), determines endothelial dysfunction and vasoconstriction, which are major factors in hypertension [102]. The mechanism and other side effects of anti-VEGF administration are not clearly known. Research underlying cardiovascular toxicity can help determine the overlapping mechanisms between cardiovascular disease and existing or future drug toxicity. To improve its management, development of guidelines for the prevention, monitoring and treatment of cardiovascular toxic DOX, it is possible that anti-VEGF/VEGFR administration might protect patients who are predisposed to cardiovascular toxicity [41].

**Fig. 1:**
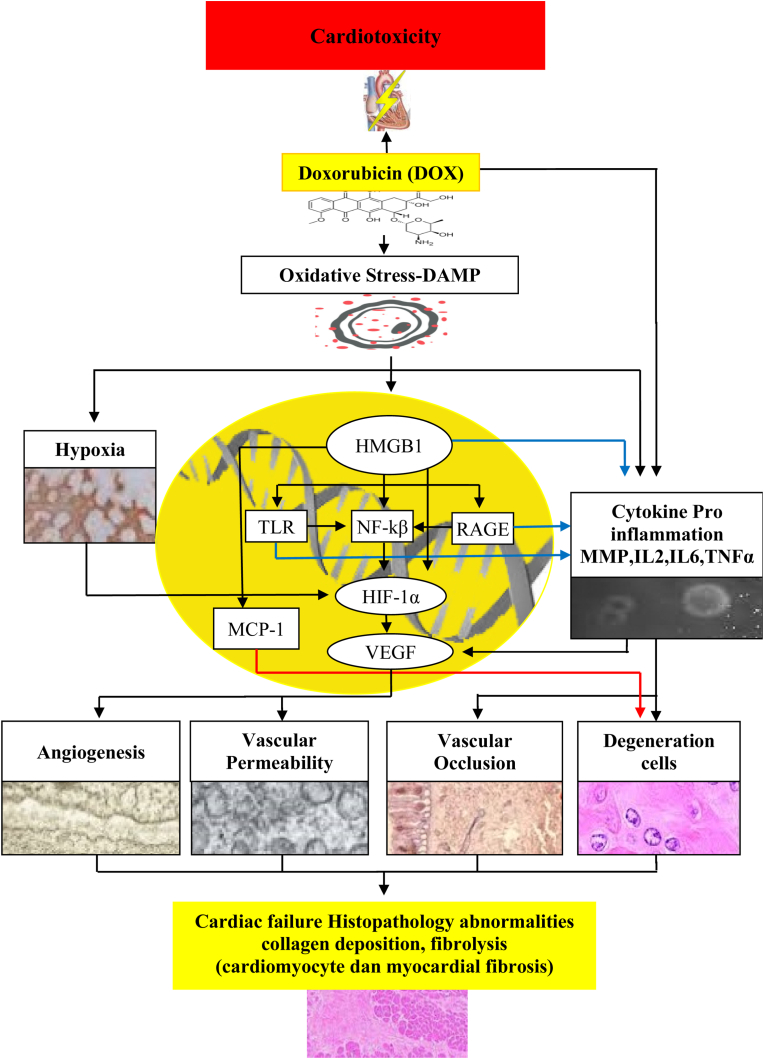
Possibility of molecular mechanisms pathway of DOX induced cardiovascular damage.

Fig. 1 shows that several literature searches may elucidate the molecular mechanisms of DOX-induced cardiovascular damage that may have a strong association with altered levels of HMGB1, HIF-1α, and VEGF in humans.

Doxorubicin (DO) is widely used for the treatment of malignancies but has side effects that can cause cardiotoxicity that can change the structure and function of the myocardium, causing severe cardiomyopathy and congestive heart failure which can lead to death. The exact causative mechanism of DOX-induced cardiotoxicity remains elusive, making it difficult to predict or prevent DOX side effects. DOX can cause oxidative stress caused by an imbalance between reactive oxygen species and endogenous antioxidants, which can lead to myocardial toxicity. In addition, DOX is an agent that can cause Associated Molecular Pattern (DAMP) damage. Oxidative stress occurs or stimulates cell surface expression of DAMP molecules, which can cause hypoxia. In addition, DOX can induce various types of proinflammatory cytokines such as IL2, IL6, and TNFα which will affect VEGF through increased HIF1 and result in angiogenesis, increased vascular permeability. Proinflammatory cytokines can also cause vascular exclusion and cell degeneration. HMGB1 triggers MMP upregulation of TLRs, of which TLR4 causes cell degeneration.

Furthermore, oxidative stress and DAMP stimulation will induce the release of HMGB1 from cells, wherein HMGB1 activates proinflammatory signaling via TLR and NFkB. The structurally diverse molecule HMGBI will bind to the RAGE multiligand receptor and will activate RAGE implying an increase in proinflammatory cytokines. Upon binding to receptors for advanced glycation end products (RAGE) or TLR4-like receptors, and HMGB1 activates vascular endothelial cells and macrophages/monocytes and regulates T (Treg) to express pro-inflammatory cytokines. Following ligand binding to RAGE, oxidative stress increases subsequently, overexpression of RAGE results in oxidative stress cycles and contributes to inflammation by up-regulation of NFκB. Activation of NFκB promotes the expression of proinflammatory cytokines, including RAGE expression, to induce prolonged activation and promotion of signaling mechanisms of cardiomyocyte damage, collagen deposition, fibrinolysis, and myocardial fibrosis through complex mechanisms such as angiogenesis, increased vascular permeability, vascular occlusion, and cell degeneration. The TLR signaling pathway culminates in the inactivation of the NFκB, which controls the expression of a range of inflammatory cytokine genes such as IL2, IL6, and TNFα. Under hypoxic conditions, it triggers the activation of HIF-1 and can interact with other enzymes and transcription factors to control vascularity, whereas activation of HIF-1, will lead to increased angiogenesis and, thus, increased oxygen supply to the area.

DOX can cause cardiotoxicity, cardiomyopathy, and congestive heart failure through inflammatory mechanisms and an increase in IL-8, NFκB, TNFα, monocyte chemotactic protein-1 (MCP-1), and Doxorubicin-mediated activation of NFκB and inflammatory cytokines by the effect of DOX through HMGB1. HMGB1 promotes fibroblast proliferation through upregulated expression of HIF1-α to induce increased VEGF. While HIF1-α is a proangiogenic transcription factor that is stabilized and activated under hypoxia. It regulates the expression of many target genes, including vascular endothelial growth factor (VEGF), and causes increased angiogenesis and vascular permeability.

## 4 Conclusion

The effect of Doxorubicin (DOX) induced cardiovascular damage may through HMGB1, HIF-1α and VEGF pathway. HMGB1, HIF-1α and VEGF has a pivotal regulator in DOX-induce cardiomyocyte damage in cardiovascular diseases which predominantly acts through different pathways. The role of HMGB1 in DOX-induced myocardial damage suggests that HMGB1 is a mediator of DOX-induced damage. In addition, DOX can inhibit HIF-1α activity where DOX can decrease HIF-1α expression and HIF-1α is also responsible for upregulation of several angiogenic factors, including VEGF. VEGF plays an important role in angiogenesis and anti-angiogenesis both in vitro and in vivo and reduces the side effects of DOX markedly. In addition, the administration of anti-angiogenesis will show an inhibitory effect on angiogenesis mediated by the VEGF signaling pathway and triggered by DOX in cells.

## Ethical approval

The article is a literature review, did not require ethical approval.

## Sources of funding

No specific funding are available.

## Author contribution

AS, BB, MH, MA, MSR, ID conceived and designed the study, conducted research, provided materials, and collected and organized data. AS, BB. MH, CK. SW, AB, and ARJ drafted the manuscript. AS, MRP, RD and MH analyzed the data and interpreted data. AS, BB, MRP, ID, SW, ARJ, AF and MH wrote initial and final draft article, and provided logistic support. All authors have critically reviewed and approved the final draft and are responsible for the content and similarity index of the manuscript.

## Trail registry number

1. Name of the registry: www.researchregistry.com
2. Unique Identifying number or registration ID: reviewregistry966
3. Hyperlink to your specific registration (must be publicly accessible and will be checked):

## Guarantor

Prof. Mochammad Hatta, MD, PhD, Clin Microbiologist (Cons).

## Provenance and peer review

Not commissioned, externally peer-reviewed.

## Declaration of competing interest

The authors declare no conflict of interest, financial or otherwise.

## Abbreviations

DAMP: = Damage-associated molecular pattern
DOX: = Doxorubicin
HIF-1α: = Hypoxia-inducible factor-1α
HMGB1: = High mobility group box protein 1
IL: = Interleukin
TNF-α: = Tumor Necrosis Factor alpha
MCP1: = Monocyte Chemoattractant Protein-1
MMP: = Matrix metalloproteinase
NFκB: = Nuclear factor kappa beta
PAMPs: = Pathogen-associated molecular patterns
RAGE: = Receptor for advanced glycation end-product
ROS: = Reactive oxygen species
STAT: = Signal transducer and activator of transcription
VEGF: = Vascular endothelial growth factor
